# Supplementary figures and images for: Vegfr3-tdTomato, a reporter mouse for microscopic visualization of lymphatic vessel by multiple modalities
Source: PLoS One. 2021 Sep 20;16(9):e0249256. doi: 10.1371/journal.pone.0249256 (PMC8452004; doi:10.1371/journal.pone.0249256)

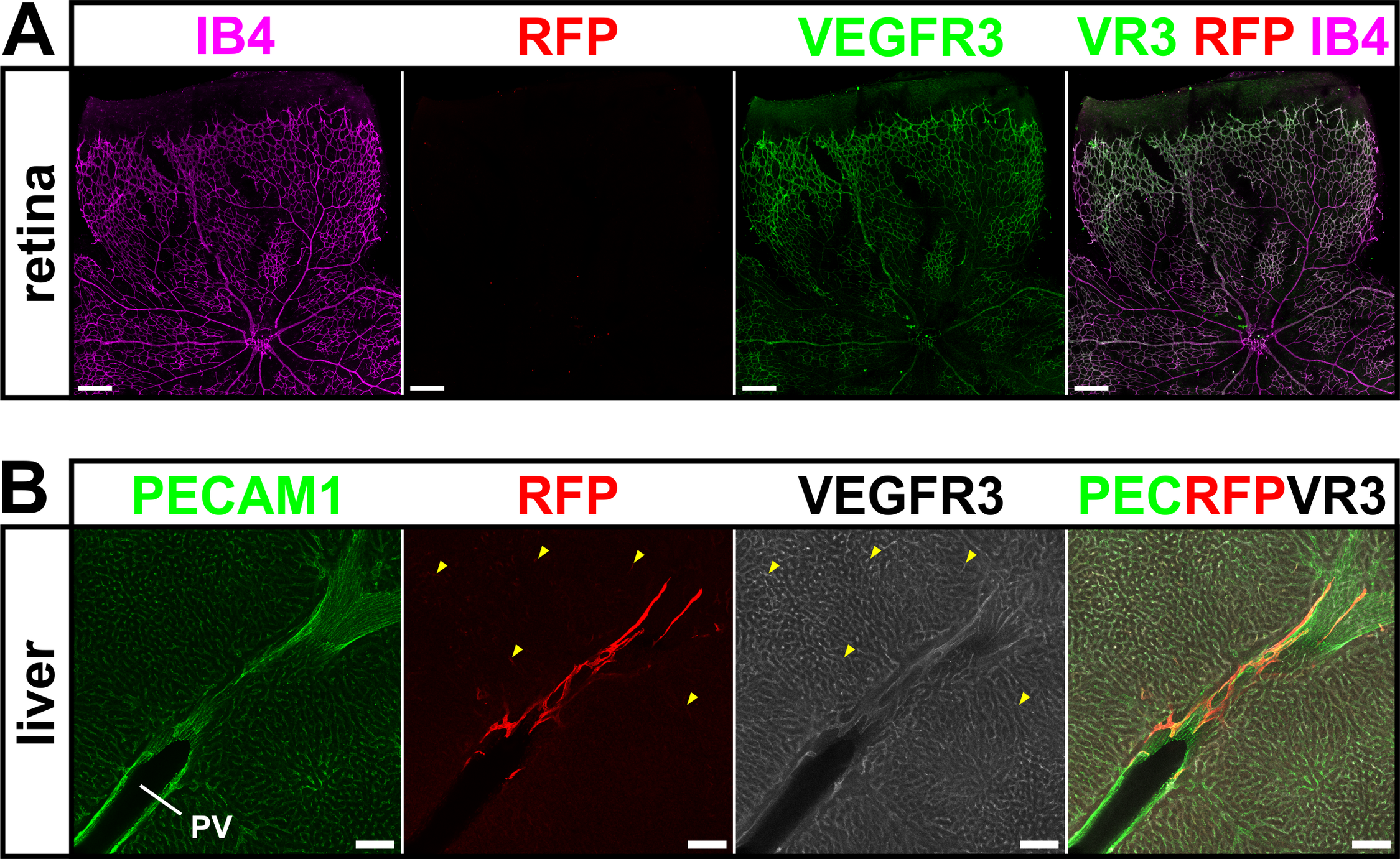

Supplement: S1 Fig — MIPs of representative confocal tile-scans from immunostained wholemount preparations of the developing retina (A) and vibratome sections of the liver (B) of postnatal Vegfr3-tdTomato transgenic mice. Stained antigens are indicated above each panel. Yellow arrow heads in B indicate VEGFR-3 and RFP double positive sinusoids. PV = portal vein. Scale bars = 300 μm (A) and 100 μm (B). (TIF) [file pone.0249256.s001.tif]

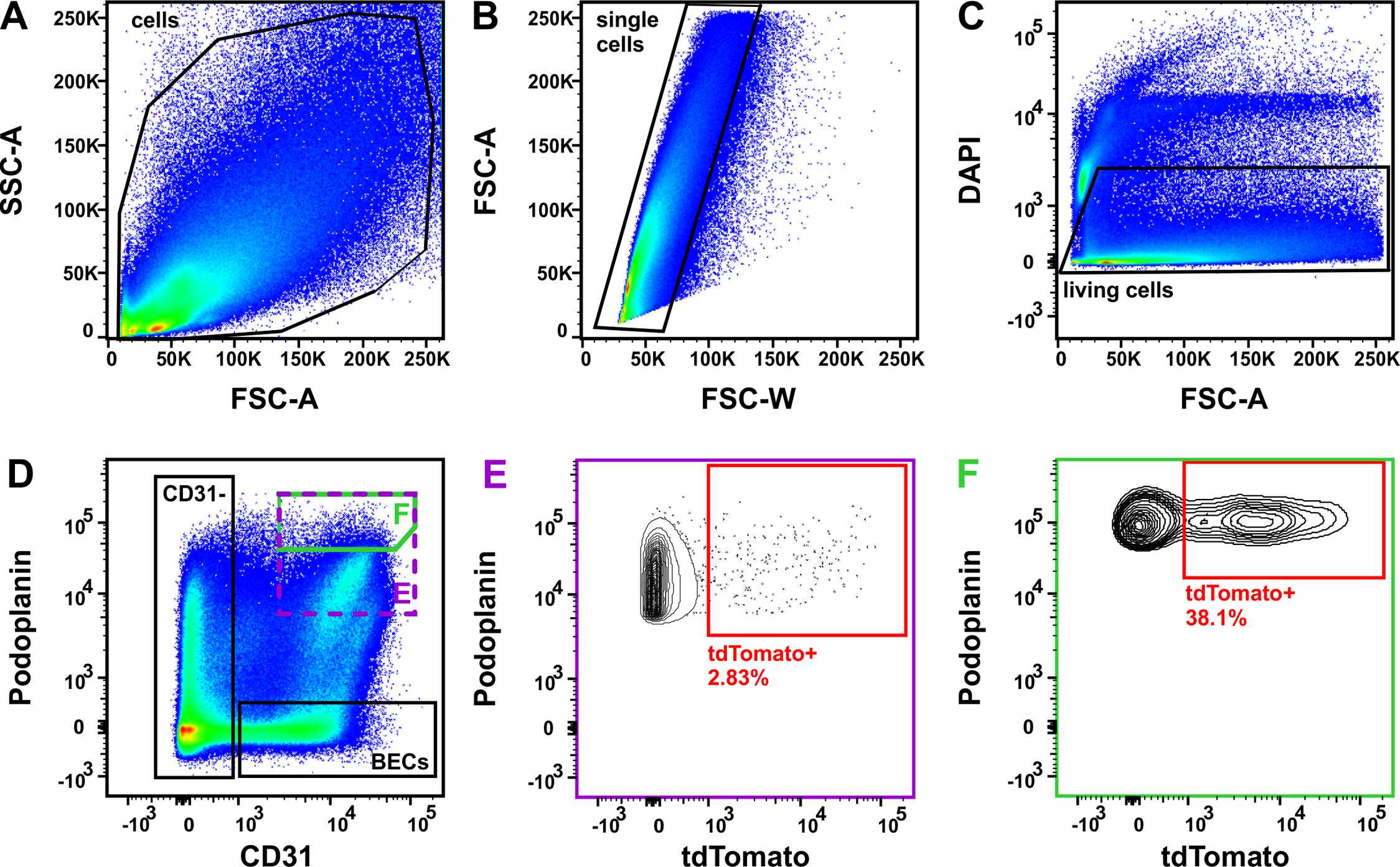

Supplement: S2 Fig — Representative FACS plots and gating scheme are shown. Isolated cells immunostained for the pan-endothelial cell marker CD31 and the common lymphatic surface marker PDPN were gated on FSC and SSC (A) to exclude cellular debris and doublets (B), and on staining with DAPI (C) to exclude dead cells. Different cell populations co-expressing CD31 and PDPN (D), were analysed for tdTomato positivity (E and F). (TIF) [file pone.0249256.s002.tif]
