# Supplementary material for: Vegfr3-tdTomato, a reporter mouse for microscopic visualization of lymphatic vessel by multiple modalities
Source: PLoS One. 2021 Sep 20;16(9):e0249256. doi: 10.1371/journal.pone.0249256 (PMC8452004; doi:10.1371/journal.pone.0249256)
Supplement: S1 Raw images — (PDF) [file pone.0249256.s006.pdf]

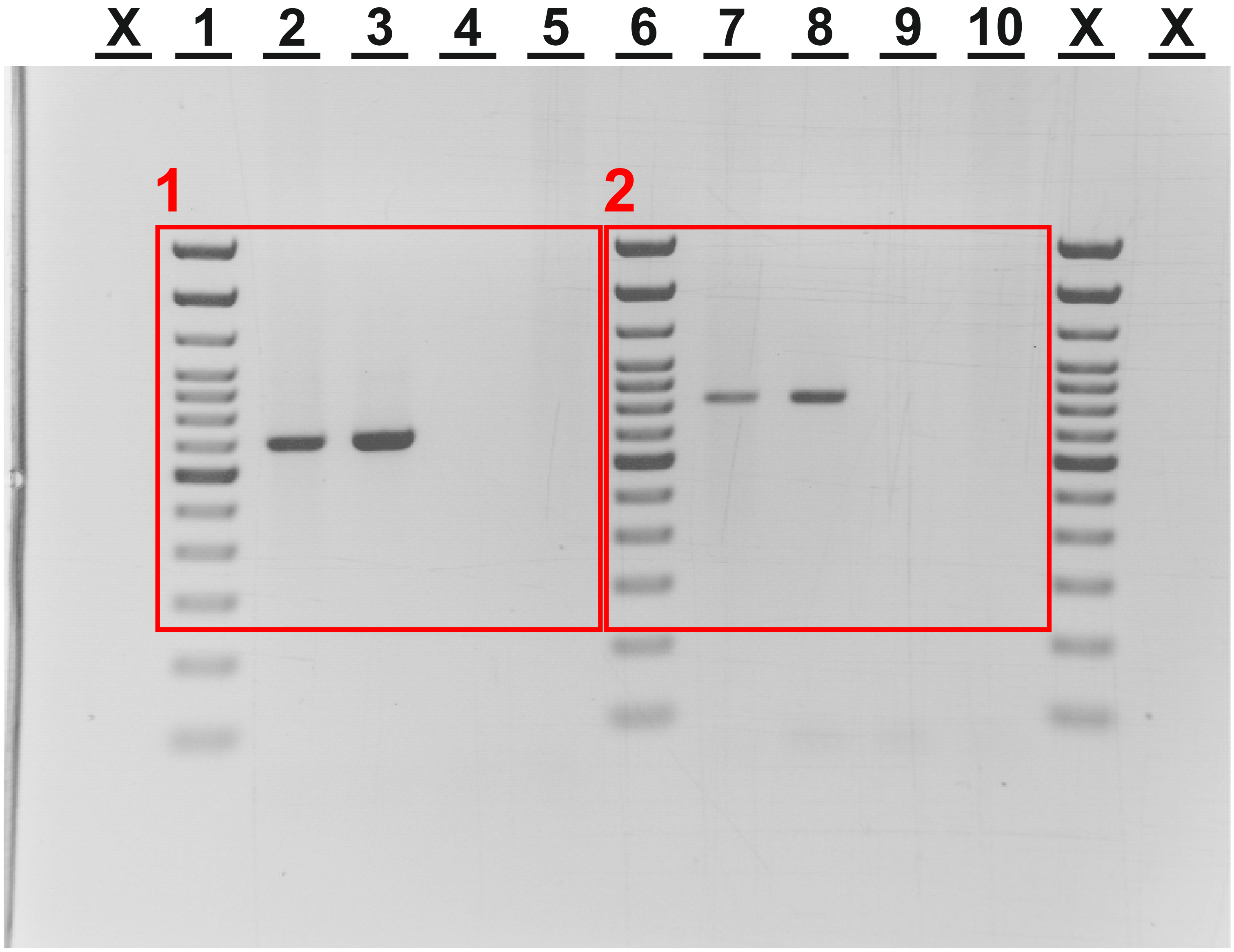

S1 raw image:
Raw gel image related to Fig 1B. Two different genotyping PCRs (1&2) were used to identify transgenic animals. Red rectangles specify the areas that were used for Fig 1B.

**Lane**	**Sample**
1/6	1kb DNA ladder
2/7	genomic DNA of Vegfr3-tdTomato$^{+/T}$ animal
3/8	modified bacterial artificial chromosome (BAC)
4/9	H_2O
5/10	genomic DNA of C57Bl/6 wildtype animal
