# Supplementary material for: Vegfr3-tdTomato, a reporter mouse for microscopic visualization of lymphatic vessel by multiple modalities
Source: PLoS One. 2021 Sep 20;16(9):e0249256. doi: 10.1371/journal.pone.0249256 (PMC8452004; doi:10.1371/journal.pone.0249256)
Supplement: Attachment — Submitted filename: Vegfr3-tdTomato_Response_to_Reviewers.docx [file pone.0249256.s007.docx]

**Detailed point by point response to the Reviewers' comments on the manuscript PONE-D-21-08111 by Redder *et al.* “*Vegfr3*-tdTomato, a reporter mouse for microscopic visualization of lymphatic vessel by multiple modalities”:**

**Review Comments to the Author**

*Reviewer #1:*

*The authors describe here a novel BAC transgenic Vegfr3-tdTomato mouse line for studying the lymphatic vasculature. By using multiple imaging modalities (confocal, 2-photon, light sheet) they highlight the technical potential of the newly described line and its application for analysis of lymphatic vessel architecture in large tissue samples. The data are convincing, with excellent quality of imaging showing lymphatic vessel labelling in multiple tissues. The line will undoubtedly become a valuable resource in the field as it provides multiple benefits compared to existing lines: for example, a single transgene driven expression of tdTomato characterized by fast maturation and exceptional brightness as well as suitability for intravital / deep tissue imaging using 2-photon microscopy due to near infrared excitation. I have just a few suggestions for consideration:*

We thank the Reviewer #1 for the positive feedback. Indeed, we believe our novel reporter line offers several advantages over already published reporter lines for the identification of lymphatic vessels and therefore will contribute to the future investigation of the lymphatic system.

*Major comments:*

*1. It would be informative to show if the reporter recapitulates the endogenous expression of Vegfr3 in a subset of BECs, for example sinusoidal ECs (e.g. liver) or tip cells of angiogenic sprouts (e.g. developing retina ).*

We thank the Reviewer for the suggestion. To analyze *Vegfr3*-tdTomato expression in these tissues, we performed wholemount staining of the explanted retina and on vibratome sections of the liver of transgenic mice. These data are included in the manuscript and we now show that the transgene was not detectable on the VEGFR-3-expressing BECs at the sprouting retinal front and only weakly labeled liver sinusoidal endothelial cells. It is therefore an excellent marker for LECs with little interference by expression on BECs, i.e. the *Vegfr3*-tdTomato transgenic mouse line is a valuable tool to study specifically lymphatic vessels.

*2. In Fig 3, it seems that some VEGFR3+LYVE1+ LECs are not positive for tdTom (or anti-RFP staining in this case). This is particularly evident in Fig 3C but can also be seen in Fig 3A. Overall, RFP signal intensity seems to be heterogenous and not necessarily correlating with VEGFR3 staining intensity. Could the authors comment on this? Flow cytometric analysis of tdTom+ LECs could be useful .*

We thank reviewer #1 for raising this important issue. As suggested, we analyzed single cell preparations from lung by cytometry. Indeed, in a CD31^pos^, Podoplanin^High^ gate that should only contain double positive bona fide LECs, still more than half of the cells did not score positive for tdTomato, based on the endogenous fluorescence of the marker protein. We presume that a fraction of the cells was genuinely not labelled because insufficient promotor activity, while some of the cells may have been missed due to insufficient sensitivity of the flow cytometer. Clearly the proportion of positive cells was higher after immune staining. We nevertheless emphasize that the reporter faithfully labels all detectable lymphatic vessels in various tissues, hence provides some beneficial lymph vessel specificity.

*Minor comments:*

*1. line 244: “… the epicardium but hardly invade into the myocardium are described for larger mammals.” I believe it is a typo and should be “… the epicardium but hardly invade into the myocardium as described for larger mammals.*

We thank the reviewer for carefully proof-reading the manuscript, the mistake has been corrected.

*Reviewer #2:*

*Redder et al report generation of Vegfr3-tdTomato transgenic mice and demonstrate utility of such model for visualization of lymphatic vessels in different organs using a variety of imaging approaches, such as confocal, light sheet and two-photon microscopy. Overall, the results are clearly presented and the manuscript is well written. The model will be a useful addition to the arsenal of tools for characterization of lymphatic vessels, especially in adult animals and pathological conditions. I have only few comments as detailed below.*

*1. It seems that a proportion of LECs does not express the reporter (e.g. Figure 3 C), could the authors quantify the proportion of such tdTomato-negative LECs using FACS?*

Quantification by flow cytometry has been performed, please see our response to reviewer #1.

*2. In addition to LECs, Vegfr3 expression has been reported in the tips of sprouting blood vessels in postnatal retina and tumors, in some fenestrated blood endothelial cells, immune cells and neural stem cells in subventricular zone of brain. Do the authors observe such pattern of expression in their model?*

We have probed expression in the tips of sprouting retinal blood vessels and liver sinusoidal endothelium, these data have now been included in the manuscript and provide string evidence for a lymph vessel specific activation of the reporter gene.

*3. The authors should discuss in more detail advantages and disadvantages of their model in comparison to other existing lymphatic reporter strains. For example, expression of Prox1 in multiple non-endothelial cell types sometimes precludes use of Prox1- based reporters.*

We thank reviewer #2 for this suggestion. We have now discussed these aspects in detail in lines 325-338 and 343-355 of the discussion, which put our *Vegfr3*-tdTomato mouse in perspective to other published reporter lines, especially those based on *Prox1* promoter expression.
